# Ornithine decarboxylase antizyme finder (OAF): Fast and reliable detection of antizymes with frameshifts in mRNAs

**DOI:** 10.1186/1471-2105-9-178

**Published:** 2008-04-02

**Authors:** Michaël Bekaert, Ivaylo P Ivanov, John F Atkins, Pavel V Baranov

**Affiliations:** 1 School of Biology and Environmental Science, University College Dublin, Belfield, Dublin 4, Ireland; 2 Bioscience Institute, University College Cork, Cork, Ireland; 3 Human Genetics Department, University of Utah, Salt Lake City, Utah, USA; 4 Biochemistry Department, University College Cork, Cork, Ireland

## Abstract

**Background:**

Ornithine decarboxylase antizymes are proteins which negatively regulate cellular polyamine levels via their affects on polyamine synthesis and cellular uptake. In virtually all organisms from yeast to mammals, antizymes are encoded by two partially overlapping open reading frames (ORFs). A +1 frameshift between frames is required for the synthesis of antizyme. Ribosomes change translation phase at the end of the first ORF in response to stimulatory signals embedded in mRNA. Since standard sequence analysis pipelines are currently unable to recognise sites of programmed ribosomal frameshifting, proper detection of full length antizyme coding sequences (CDS) requires conscientious manual evaluation by a human expert. The rapid growth of sequence information demands less laborious and more cost efficient solutions for this problem. This manuscript describes a rapid and accurate computer tool for antizyme CDS detection that requires minimal human involvement.

**Results:**

We have developed a computer tool, OAF (ODC antizyme finder) for identifying antizyme encoding sequences in spliced or intronless nucleic acid sequenes. OAF utilizes a combination of profile hidden Markov models (HMM) built separately for the products of each open reading frame constituting the entire antizyme coding sequence. Profile HMMs are based on a set of 218 manually assembled antizyme sequences. To distinguish between antizyme paralogs and orthologs from major phyla, antizyme sequences were clustered into twelve groups and specific combinations of profile HMMs were designed for each group. OAF has been tested on the current version of dbEST, where it identified over six thousand Expressed Sequence Tags (EST) sequences encoding antizyme proteins (over two thousand antizyme CDS in these ESTs are non redundant).

**Conclusion:**

OAF performs well on raw EST sequences and mRNA sequences derived from genomic annotations. OAF will be used for the future updates of the RECODE database. OAF can also be useful for identifying novel antizyme sequences when run with relaxed parameters. It is anticipated that OAF will be used for EST and genome annotation purposes. OAF outputs sequence annotations in fasta, genbank flat file or XML format. The OAF web interface and the source code are freely available at  and at a mirror site .

## Background

Ornithine Decarboxylase Antizymes are important negative regulators of cellular polyamine levels. In mammals, antizyme-1 inhibits ornithine decarboxylase (ODC), an enzyme catalyzing the first and rate-limiting step in polyamine biosynthesis. Antizyme-1 binds to ODC and targets it for ubiquitin-independent degradation by the 26S proteosome in a multiple-turnover manner (a single antizyme molecule can cause degradation of several ODC molecules) [1,2]. Additionally, antizyme-1 regulates the intracellular concentration of polyamines by inhibiting cellular import of polyamines and accelerating polyamine export from the cell [3-5]. While genomes of lower eukaryotes contain single antizyme genes, multiple paralogs have evolved in higher eukaryotes, with at least two antizymes in vertebrates [6,7], three in mammals [8,9] and up to five in certain fish species [10]. Antizyme paralogs vary somewhat in their function, although all are implicated in the regulation of polyamine synthesis (and some are reported to link with other pathways [11,12]). Antizyme paralogs usually have a distinct expression pattern with certain paralogs being expressed in a strictly restrictive tissue-specific manner, such as testis-specific mammalian antizyme 3 [8,9] or retina and brain specific antizyme AZR from *Danio rerio *[13]. Reviews of antizyme function and distribution are available [10,14,15].

**Figure 1:**
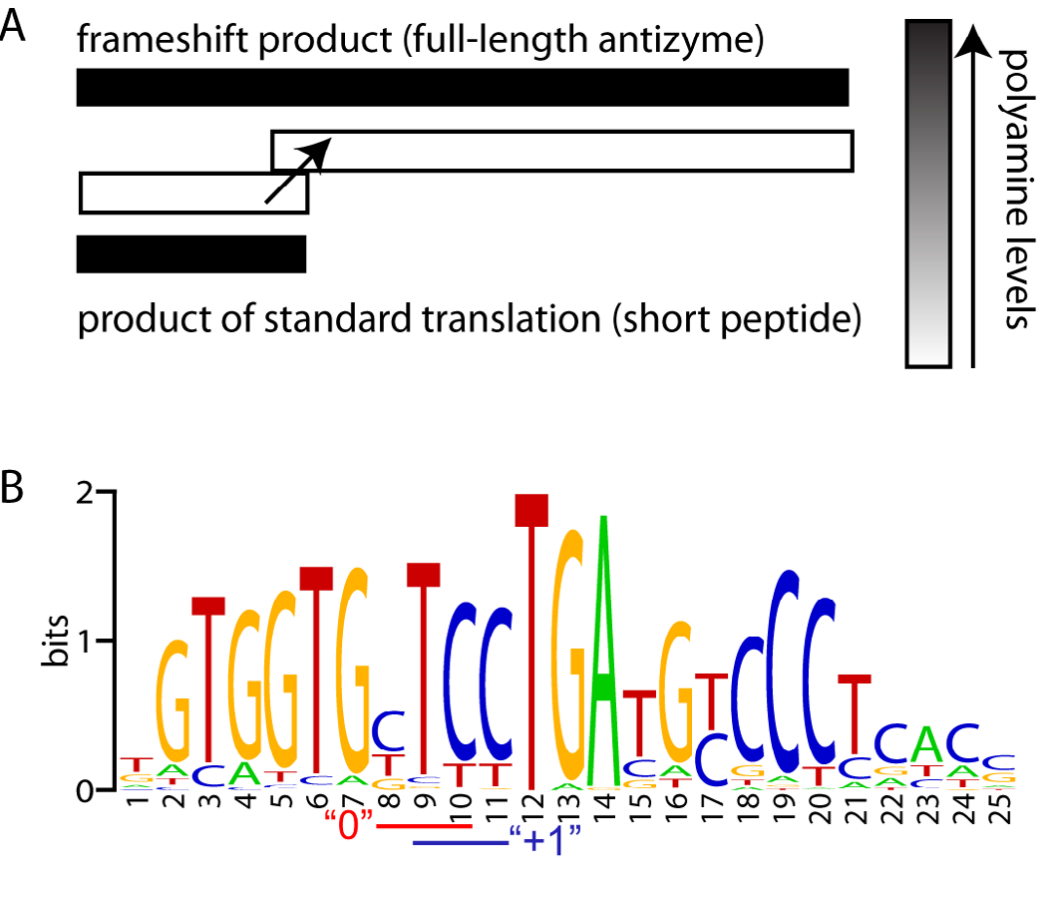
**Scheme of negative regulatory feedback in regulation of antizyme synthesis and conservation of the frameshift site**. **A**. The competition between ribosomal frameshifting and standard translation (termination) is sensitive to polyamine levels. An increase in polyamine concentrations shifts the competition toward frameshifting resulting in elevation of antizyme synthesis and consequent inhibition of polyamine synthesis and uptake. A decrease in polyamine concentrations shifts the competition towards standard translation and produces the opposite effect on the synthesis of antizyme and polyamines. **B**. WebLogo representing the alignment of 153 OAZ sequences. The last codon in the zero frame and the first codon -in +1 frame are indicated by red and blue bars respectively. It can be seen that the only universal nucleotide of the frameshift site is T (U in mRNA) corresponding to the first position of the stop codon at the end of the first ORF.

Given the important role that antizymes play in the regulation of polyamine concentrations, it is not surprising that their own biosynthesis is regulated in response to changes of cellular polyamine concentrations. Polyamines' concentrations are sensed during the elongation stage of antizyme mRNA translation. Unlike the great majority of CDS-es, that for virtually all eukaryotic antizymes consists of two overlapping open reading frames. Synthesis of full-length antizyme protein requires a portion of translating ribosomes to switch translation phase at the end of the first ORF into the partially overlapping ORF (in +1 translation phase) in a process termed programmed ribosomal frameshifting [16]. The portion of ribosomes that do not shift frames, terminate at the end of the first ORF with release of relatively short encoded polypeptide. Increases in cellular polyamine levels result in elevated frameshifting efficiency and so of synthesis of fully functional antizyme. The competition between frameshifting and termination at the end of the first ORF is a sensor of polyamine concentration that provides an elegant mechanism for regulatory negative feedback (Figure 1A).

The +1 frameshifting event during antizyme biosynthesis significantly complicates automatic detection of its full-length CDS in mRNA. This is due to the lack of reliable and efficient algorithms for predicting ribosomal frameshifting locations. A number of attempts have been made recently to develop computational approaches for predicting instances of the ribosomal frameshifting [17-22]. Some of these approaches could be useful for detecting candidate sequences that are prone to efficient (not necessarily programmed) frameshifting within particular groups of organisms [17-19,23]. However, they are not suitable for reliable detection of programmed ribosomal frameshifting events without experimental verification or additional expert human involvement. The reasons underlying the consistent failure to develop highly accurate algorithms for ribosomal frameshifting prediction lie in the very nature of programmed ribosomal frameshifting. The efficiency of ribosomal frameshifting is modulated by highly diverse sequence elements many of which evolved independently. The mechanisms by which such elements alter translation also vary considerably. The situation is further complicated by differences in the translation machinery (sequences of ribosomal components, differences in tRNAs properties and their relative concentrations) across different organisms, leading to a situation where the same sequence is shift-prone in one organism, but in another it is accurately translated in a standard triplet-manner. Therefore, it is not possible to find even a single nucleotide sequence feature that would specify a site of ribosomal frameshifting universal for all organisms. Information regarding the diversity of genes utilizing programmed ribosomal frameshifting for their expression as well as multifarious sequences modulating frameshifting process is available at the Recode database, which is currently the richest Internet resource [24,25], as well as, comprehensive literature reviews on this and related topics [26-35]. In fact, currently antizyme mRNAs themselves are the most plentiful source of diverse frameshift stimulator signals as evident from the recent detailed review covering nearly three hundred antizyme mRNA sequences [10]. A collection of sequences described in that review was used here for the design of OAF (Additional file 1).

It appears that approaches to predict frameshifting specifically for particular clusters of related genes produce more reliable results. Such approaches were applied for -1 frameshifting involved in the synthesis of viral polyproteins [21], different types of frameshifting events in decoding bacteriophage tail assembly genes [20], and +1 frameshifting during the synthesis of bacterial release factors 2 [22]. Indeed ribosomal frameshifting utilized by a group of homologous genes likely has the same origin. While evolution introduces organism specific alterations in the sequence of the frameshifting cassette, as well as, diversifying protein sequence, a detectable degree of similarity is frequently recognizable. Though existence of such similarity may not be a universal rule (as evident with the frameshifting utilized in decoding bacteriophage tail assembly genes [20] where only genomic localization of overlapping ORFs is conserved), it holds true for many cases. Therefore, knowledge of a few examples of ribosomal frameshifting from homologous genes can be sufficient for designing algorithms for automatic and accurate prediction of ribosomal frameshifting utilized in decoding of homologous genes. By dealing with each group of homologous genes utilizing ribosomal frameshifting separately one-by-one, we aim to build a collection of autonomic computer tools capable of automatically predicting most cases of ribosomal frameshifting in newly sequenced organisms. OAF is our second computer tool designed in pursuit of this goal. Our first tool, ARFA detects and annotates the programmed ribosomal frameshifting required for expression of certain bacterial release factors [22]. Both tools will be used for future updates of the Recode database.

## Implementation

OAF is written in Perl, it utilizes BioPerl libraries [36]. The OAF Web interface was designed using PHP.

### Outline of the analysis performed by OAF

**Figure 2:**
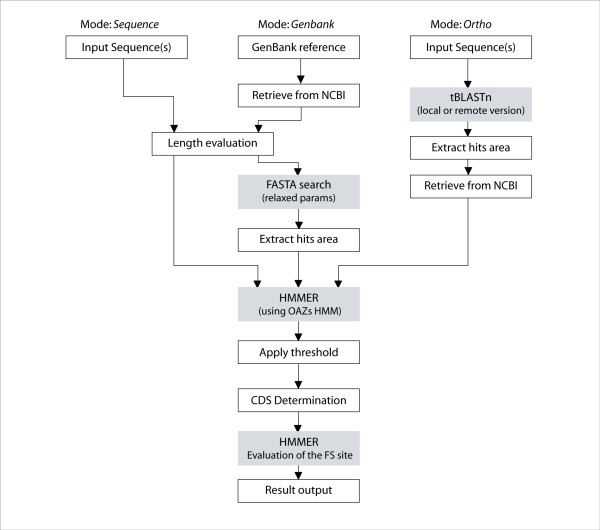
**Scheme of analyses performed by OAF**. OAF pipeline. Each step performed by OAF is shown as a box, grey boxes represent external modules utilized by OAF.

Antizyme mRNAs from different organisms have evolved a remarkable assortment of RNA signals for stimulating or modulating the +1 ribosomal frameshifting used in their expression. Many sequence features are shared among closely related antizyme mRNAs. For example, two distinct types of frameshift-enhancing RNA pseudoknots are embedded in antizyme-1 and antizyme-2 mRNAs from mammals. Nevertheless, not a single feature is universally conserved. Instead of trying to account for known frameshifting stimulators, we have devised an antizyme gene detection scheme based on detection of sequences encoding antizymes. While antizyme protein sequences are highly diverse, there is a reasonable degree of sequence similarity within large phylogenetic groups allowing their detection based on similarity searches. Most importantly, eukaryotic antizyme genes share the same ORF organisation: the upstream ORF is smaller than the downstream ORF and the downstream ORF is always in the +1 translational phase relative to the first one. Therefore our method is based on a search for two overlapping ORFs corresponding to profile HMMs designed using sequences of known antizymes. Mutual orientation of the ORFs is further examined to verify that it corresponds to an expected transition between translational phases. For large sequences (>20 kb), OAF performs an initial FASTA search with relaxed parameters, where a mixture of divergent antizyme sequences is used as a query. This is used to increase OAF speed by reducing the number of candidate sequences for subsequent HMM analysis. Relaxed parameters decrease the chances of losing true positives in this process. The scheme of analyses performed by OAF is illustrated in Figure 2.

### Profile HMMs and automatic classification of antizymes

**Figure 3:**
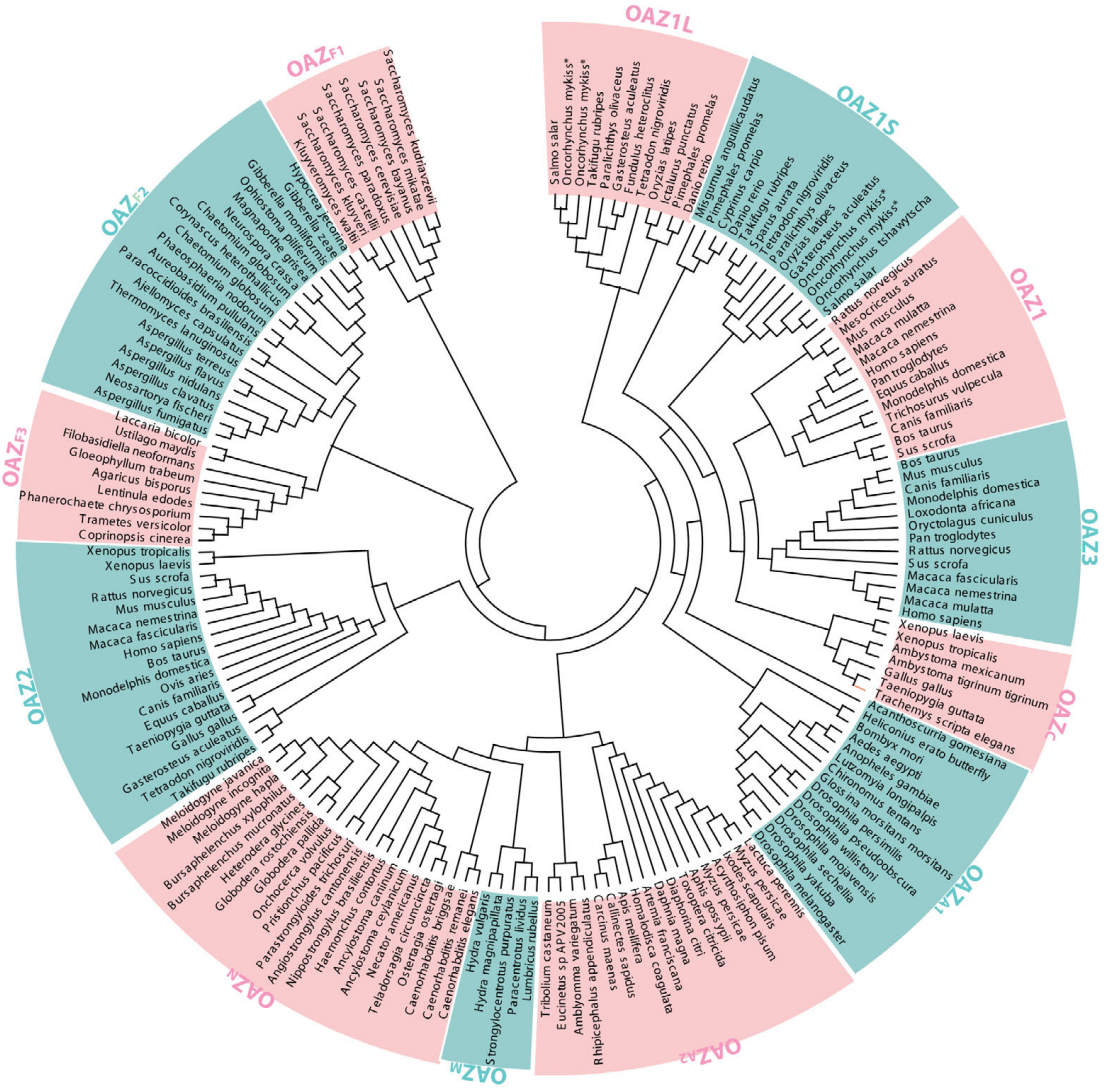
**OAZ clustering**. A circular tree of OAZ sequences representing clustering that has been used to design profile-HMMs used by OAF.

To design profile HMMs exploited by OAF, we used a collection of protein sequences derived from mRNA fragments using manually assembled ESTs. These sequences were described in some detail in a recent antizyme review [10] and are available in this article as an Additional File 1 (manualOAZs.fasta). Evolutionary distances between protein sequences were estimated using a Neighbour-Joining algorithm and poisson correction evolutionary model implemented in MEGA3.1 program [37]. Based on these distances, sequences were clustered into 12 homologous groups for which separate pairs of profile HMMs were designed using HMMER [38]. These HMMs are used to allow discrimination among different antizyme paralogs and to permit approximate estimation of the taxonomic origins of antizyme encoding sequences. The clustering is shown on the tree generated with MEGA3.1 (see Figure 3).

A separate profile HMM is built for the frameshift site itself. This HMM is not used for identification of antizymes or frameshift sites. However a predicted frameshift site is compared to the HMM and corresponding E-score can be reported in the output to facilitate further processing of data such as identification of unusual frameshift sites or detection of sequencing errors disguised as cryptic frameshift sites. Figure 1B illustrates conservation of OAZ frameshift sites as a web logo [39].

### OAF I/O interface

#### Input

There are two types of searches that can be performed by OAF. First a given nucleotide sequence or multiple sequences (either provided in a user's file in a fasta format or as a Genbank accession number) can be analyzed for the presence of antizyme CDS (first two modes in Figure 2). Second (third mode in Figure 2), protein sequences of known antizymes in a user's fasta file can be used as query for a search against a database of nucleotide sequences (either in a local Blast database or in a remote BLAST database at NCBI). A user can specify the genetic code table and usage of alternative initiation codons (by default CDS can start only with ATG/AUG).

#### Output

OAF reports sequences of encoded antizymes either as raw sequence, or in fasta, genbank or XML format. XML contains detailed information regarding the frameshift site and is compatible with a future version of Recode database. By default, OAF reports all sequences encoding antizymes, even if their ORF organization does not correspond to that for utilization of +1 frameshifting or if only a partial antizyme sequence is found. Such, likely erroneous sequences, can be filtered out automatically.

### Web interface

The web interface of OAF (see Availability and Requirements section). It serves mostly illustrative purposes and has limited capabilities compared to a full version of Oaf. Web service allows analysis of a single user-provided sequence for the presence of encoding antizyme.

## Results and Discussion

To evaluate OAF prediction sensitivity for genome annotations, the mRNA sequences of 20 completed eukaryotic genomes were downloaded from the RefSeq database [40]. OAF detected 18 OAZ genes (Table 1). No genes encoding antizymes were detected in plant genomes (Table 1). To evaluate OAF prediction selectivity, a random sequence database (totalling 10 Tbp) was generated by a fifth order Markov chains based on six-mer frequencies of each mRNA of the genomic sequences. OAF did not detect any OAZ sequence in this database.

**Table 1:** OAZ sequences detected in completed genomes

**Species**	**Expected**	**Predicted**
*Anopheles gambiae*	1	1
*Arabidopsis thaliana*	0	0
*Caenorhabditis elegans*	1	1
*Drosophila melanogaster*	1	1
*Encephalitozoon cuniculi*	?	1
*Guillardia theta nucleomorph*	0	0
*Saccharomyces cerevisiae*	1	1
*Plasmodium falciparum*	?	0
*Schizosaccharomyces pombe*	1	1
*Homo sapiens*	3	3
*Mus musculus*	3	3
*Rattus norvegicus*	3	3
*Danio rerio*	3	3
*Avena sativa*	0	0
*Glycine max*	0	0
*Hordeum vulgare*	0	0
*Lycopersicon esculentum*	0	0
*Oryza sativa*	0	0
*Triticum aestivum*	0	0
*Zea mays*	0	0

OAZ genes in completed genomes. Organism names are given in the first column. The second column shows the number of OAZ genes that expected in the corresponding genome prior to the analysis. The third column shows a number of OAZ genes found by OAF.

To estimate OAF accuracy on EST sequences, the June 2007 dbEST was used [41]. OAF detected antizyme sequences in 6639 ESTs, among them there are 2067 unique sequences coding for antizyme. Many of these sequences were truncated mRNA fragments that can be grouped as corresponding to the same antizyme mRNA. 24 new antizyme sequences, which were not present in the original dataset (Additional file 1), were detected, see Table 2.

**Table 2:** New OAZ sequences detected in dbEST

**Species**	**OAZ cluster**	**EST**
*Amblyomma cajennense*	OAZ (A2)	EC778732 EC779052 EC779122
*Ambystoma mexicanum*	OAZ1	CN034176 CN035835 CN036385 CN036992 CN037270 CN037900 CN038182 CN039093 CN039394 CN041555 CN043386 CN045230 CN045468 CN045566 CN045877
*Ambystoma mexicanum*	OAZ1	CN034176 CN035835 CN036385 CN036992 CN037270 CN037900 CN038182 CN039093 CN039394 CN041555 CN043386 CN045230 CN045468 CN045566 CN045877
*Biomphalaria glabrata*	OAZ (M)	ES491521
*Bursaphelenchus mucronatus*	OAZ (N)	CJ977611 CJ978020 CJ978332
*Bursaphelenchus xylophilus*	OAZ (N)	CJ979174 CJ980047 CJ980437 CJ981142 CJ981263 CJ981361 CJ981516 CJ982573 CJ984323 CJ985769 CJ985981 CJ986817 CJ987169 CJ987483 CJ987938 CJ989363 CJ990870
*Chlamys farreri*,	OAZ (M)	DT716986
*Gadus morhua*	OAZ1L	ES475944
*Gadus morhua*	OAZ1S	ES479153
*Gammarus pulex*	OAZ (A2)	EH276450
*Haliotis asinina*	OAZ (M)	DW986242 DY402940
*Heterorhabditis bacteriophora*	OAZ (N)	ES408646 ES409780 ES410567 ES411071 ES411545 ES412181 ES413997 ES414281
*Hypocrea virens*	OAZ (F2)	EH628788
*Idiosepius paradoxus*	OAZ (M)	DB912841 DB914374 DB915016 DB916854 DB918091 DB918471 DB918744 DB919717 DB920015
*Lipochromis sp. *'matumbi hunter'	OAZ1L	DB859357 DB860568 DB862111 DB862372 DB864801 DB870209 DB873104
*Lysiphlebus testaceipes*	OAZ (A1)	EH012818 EH014124 EH015343 EH016344 EH016850
*Meleagris gallopavo*	OAZ2	EH286700 EH293671
*Meloidogyne arenaria*	OAZ (N)	BI747416
*Ornithodoros parkeri*	OAZ (A2)	ES581004
*Osmerus mordax*	OAZ2	EL518444
*Osmerus mordax*	OAZ1L	EL539625
*Poecilia reticulata*	OAZ1L	ES373335 ES373822 ES376344 ES380163
*Poecilia reticulata*	OAZ1S	ES384943
*Rhodnius prolixus*	OAZ (A1)	EH114285

OAZ sequences found in dbEST which were not present in the original dataset of OAZ protein sequences. Organism names are given in the first column, the second column shows OAF clustering of OAZ sequences. The third column lists EST accession numbers.

OAF has detected a number of highly similar variant OAZ sequences supported by multiple ESTs corresponding to the same species. Some of these sequences are most likely allelic variants while others correspond to recent gene duplication events. OAZ variants are summarized in Table 3.

**Table 3:** OAZ sequence variants in dbEST

Species	OAZ type	EST	Complete	Partial	Origin
Argopecten irradians*	OAZ (M)	CF197657	1	2	Allele
Argopecten irradians*	OAZ (M)	CK484346	1	2	Allele
*Danio rerio*	OAZ1S	CA787369	27	3+	Allele
*Danio rerio*	OAZ1S	CD760042	57	3+	Allele
Gasterosteus aculeatus*	OAZ1S	DN717815	15	3+	Allele
Gasterosteus aculeatus*	OAZ1S	DN722004	8	3+	Allele
Hydra magnipapillata*	OAZ (M)	BP515897	3	3+	Allele
Hydra magnipapillata*	OAZ (M)	DT613005	5	3+	Allele
*Macaca nemestrina*	OAZ1	DR772378	2	3+	Allele
*Macaca nemestrina*	OAZ1	EB520932	2	3+	Allele
*Mus musculus*	OAZ2	BQ713320	7	3+	Allele
*Mus musculus*	OAZ2	CB196396	6	3+	Allele
Oncorhynchus mykiss*	OAZ1L	BX856931	1	1	Duplication
Oncorhynchus mykiss*	OAZ1L	CA376808	0	2	Duplication
Oncorhynchus mykiss*	OAZ1S	CA348102	4	3+	Allele
Oncorhynchus mykiss*	OAZ1S	CA357487	1	3+	Allele
Oncorhynchus tshawytscha*	OAZ1S	CA345360	0	3+	Duplication
Oncorhynchus tshawytscha*	OAZ1S	CX352386	1	2	Duplication
Pimephales promelas*	OAZ1L	DT246851	7	3+	Allele
Pimephales promelas*	OAZ1L	DT260869	4	3+	Allele
Pimephales promelas*	OAZ1L	DT262889	2	3+	Allele
Pimephales promelas*	OAZ1L	DT346263	3	3+	Allele
Pimephales promelas*	OAZ1L	DT359488	2	3+	Allele
Pimephales promelas*	OAZ2	DT117815	6	3+	Allele
Pimephales promelas*	OAZ2	DT135205	4	3+	Allele
*Rattus norvegicus*	OAZ2	CK366765	6	3	Allele
*Rattus norvegicus*	OAZ2	CV105321	2	3	Allele
Salmo salar*	OAZ1S	DW536698	7	3+	Duplication
Salmo salar*	OAZ1S	DY726570	3	3+	Duplication
Taeniopygia guttata*	OAZ2	DV947965	1	2	Allele
Taeniopygia guttata*	OAZ2	DV961695	1	2	Allele

Summary of OAZ sequence variants. Names of organisms are shown in the first column. The second column shows OAZ classification according to OAF clustering. The third column lists accession numbers for representative ESTs. The fourth column shows a number of EST sequences containing complete OAZ CDS. The fifth column shows a number of ESTs containing a partial OAZ CDS. Likely reasons for the existence of OAZ variants are given in the last column. Asterisks indicate no complete genome sequence for the corresponding organism, meaning that our assessment of variant origin is presumptive.

OAF detected a number of sequences whose OAZ clustering (Figure 3) did not match the taxonomy of the source organisms. These sequences are likely contaminants that were introduced from pests, symbionts, food or cell hosts (see Table 4). Some of these contaminations were previously reported in [10].

**Table 4:** Contaminant OAZ sequences

**Species**	**Family**	**Expected cluster**	**Found cluster**	**Family of the best Blast hit**	**Possible source of contamination**	**EST**
*Toxoplasma gondii*	*Sarcocystidae*	-	OAZ1	*Suidae*	From cell host (pig)	DV107889 DV107991
*Citrus clementina*	*Rutaceae*	-	OAZ (A1)	*Cicadillidae*	From pest (sharpshooter?)	DY278292
*Populus tremula × Populus tremuloides*	*Salicaceae*	-	OAZ (A1)	*Aphididae*	From pest (aphid?)	BU826038 DN500296
*Lactuca perennis*	*Asteraceae*	-	OAZ (A1)	*Aphididae*	From pest (aphid?)	DW084698
*Pinus taeda*	*Pinaceae*	-	OAZ (F1)	*Corticiaceae*	From symbiont fungus	CF389943
*Danio rerio*	*Cyprinidae*	OAZ1L/OAZ1S/OAZ2	OAZ (A1)	*Acrididae*	From food (Brian shrimp?)	CK868153

OAZ corresponding to sequences from contaminating organisms are shown. Names of organisms where contaminants were found are given in the first column. The second column represents the corresponding taxonomic family. The third column shows an OAZ cluster to which an OAZ is expected to belong based on the taxonomy of the source organism. The third column shows a cluster to which the sequence belongs. The fourth column lists the likely source of contamination. The fifth contains EST accession numbers.

## Conclusion

We have developed a simple computer utility for identification of OAZ encoding sequences in nucleic acids, called OAF (ODC antizyme finder). It performs with high speed and accuracy on mRNA sequences annotated in completed genomes as well as on raw RNA sequences from EST collections.

## Availability and requirements

* Project name: OAF (Ornithine Decarboxylase Antizyme Finder)

* Project home pages: 



* Operating system(s): Platform independent

* Programming language: Perl, PHP

* Other requirements: Mandatory: BioPerl 1.5.1+, FASTA 3.4+, HMMER 2.3.2. Optional (required for searches against local blast databases): NCBI BLAST

* License: CCL

* Any restrictions to use by non-academics: yes, see the home page.

## Abbreviations

ARFA: Automatic Release Factor Annotation tool; AZR: AntiZyme from Retina; BLAST: Basic Local Alignment Search Tool; CDS: CoDing Sequence; EST:  Expressed Sequence Tag; HMM: Hidden Markov Model; MEGA: Molecular Evolution Genetic Analysis; mRNA: messenger RiboNucleic Acid; NCBI: National Center of Biotechnology and Informatics, Perl: Practical Extraction and Report Language; PHP: Personal Home Page tools; OAF: Ornithine decarboxylase Antizyme Finder; OAZ: Ornithine decarboxylase AntiZyme; ODC: Ornithine DeCarboxylase; ORF: Open Reading Frame; RNA: RiboNucleic Acid; tRNA: transport RiboNucleic Acid; URL: Uniform Resource Locator; XML: eXchange Markup Language.

## Competing interests

The authors declare that they have no competing interests.

## Authors' contributions

MB designed and scripted OAF and its web interface. IPI manually reconstructed antizyme mRNA sequences from EST collections. JFA provided encouragement, general coordination and financial support to the project. PVB conceived the project, helped to design OAF and wrote the manuscript. All authors have contributed to the final revision of the manuscript.

## Supplementary Material

Additional file 1 Collection of OAZ sequences. Manually collected and assembled OAZ sequences that were used to device OAF. Click here for file
